# Differentiation in water adaptation strategy between epiphytic and terrestrial species of *Cymbidium*, Orchidaceae

**DOI:** 10.1093/aobpla/plaf030

**Published:** 2025-06-02

**Authors:** Tian-Yang Gao, Wei Zhang, Shi-Bao Zhang

**Affiliations:** Key Laboratory for Economic Plants and Biotechnology, Kunming Institute of Botany, Chinese Academy of Sciences, Kunming, Yunnan 650201, China; Yunnan Key Laboratory for Wild Plant Resources, Kunming, Yunnan 650201, China; University of Chinese Academy of Sciences, Beijing 100049, China; Key Laboratory for Economic Plants and Biotechnology, Kunming Institute of Botany, Chinese Academy of Sciences, Kunming, Yunnan 650201, China; Key Laboratory for Economic Plants and Biotechnology, Kunming Institute of Botany, Chinese Academy of Sciences, Kunming, Yunnan 650201, China; Yunnan Key Laboratory for Wild Plant Resources, Kunming, Yunnan 650201, China; Form & Function

**Keywords:** *Cymbidium*, life form, root trait, leaf trait, water adaptation

## Abstract

The contrasting water availabilities of epiphytic and terrestrial habitats likely drive divergent adaptive strategies for water use in plants. While leaf adaptations are well studied, the role of root traits in water adaptation remains poorly understood. Here, we compared the leaf and root anatomical traits of 9 terrestrial and 15 epiphytic species within *Cymbidium* (Orchidaceae) using a common garden experiment. Our results demonstrated that terrestrial species exhibited leaves with larger stomata and roots with enhanced cortical development, whereas epiphytes possessed thicker velamen radicum and larger xylem vessels. Correlation analyses revealed coordinated leaf–root trait relationships: in epiphytes, reduced velamen thickness/radius correlated with increased cortical investment, along with a positive correlation between stomatal density and cortex-related traits, while terrestrial species exhibited a strong association between leaf thickness and xylem vessel dimensions. These results revealed significant differences in leaf and root traits between epiphytic and terrestrial species. Epiphytic species exhibited greater potential for drought tolerance, while terrestrial species showed more resistance to waterlogging stress. These findings provide important insights into the water-adaptation strategies of orchids.

## Teaser

Orchids exhibit several life forms, which may employ different water adaptation strategies. In this study of 24 Cymbidium species, the authors find that epiphytic orchids tolerate drought stress by deceasing water loss and increasing water transport, while terrestrial orchids adapt to waterlogged soils by building extensive air-filled root chambers with enhanced cortex. The findings are important for understanding the ecological adaptation of orchids and promoting species conservation.

## Introduction

With nearly 800 genera and over 29 000 species, the Orchidaceae, world-renowned ornamental and medicinal plants, is one of the largest and most diverse families of flowering plants (Christenhusz and Byng 2016, Govaerts et al. 2021). Approximately 70% of species in this family are epiphytic (Benzing 1987). Epiphytes grow on the canopies of other plants and basically rely on the water and nutrient supplies from the materials around their roots or the atmosphere to survive (Rasmussen and Rasmussen 2018). Previous studies indicated that epiphytic habitats provide diversified niches, which contribute to species differentiation and speciation (Benzing 1987). However, the limited and irregular moisture availability in epiphytic habitats becomes the primary limiting factor for the growth and establishment of epiphytes (Laube and Zotz 2003).

Variations in plant morphological and anatomical traits can reflect the resource acquisition strategies and are closely related to their physiological functions and stress tolerances (Guan et al. 2010, Gifford et al. 2013, Wang et al. 2017, Zhang et al. 2018, Ramírez-Valiente et al. 2019, Li et al. 2020). Previous studies on orchids have focused on leaf traits, such as those in *Dendrobium* (Sun et al. 2014), *Paphiopedilum*, *Cypripedium* (Guan et al. 2010), and *Cymbidium* (Zhang et al. 2015). Root physiological traits can be strongly influenced by anatomical features, which affect the balance between acquiring and conserving resources, ultimately impacting the distribution of plant species (Pedersen et al. 2020, Yamauchi et al. 2020). For example, gymnosperms have a higher stele and lower cortex proportion, favouring higher root transport capacity to maintain root survival in dry and cold environments (Guo et al. 2008). Enhanced drought tolerance in upland rice is linked to the presence of expanded xylem vessels and thicker roots (Gowda et al. 2011). However, as the key organ for water absorption, the function of root traits in water adaptation of epiphytes is less understood.

The whole-plant economic spectrum hypothesis suggested that leaf and root traits might evolve coordinately. Previous studies have found that many traits of these two organs are interconnected (Craine et al. 2005, Tjoelker et al. 2005, Freschet et al. 2010, Vleminckx et al. 2021, Zhou et al. 2023). Understanding these associations helps to elucidate the mechanisms of interactions between plant traits and resource use and allocation during plant growth, whereas a previous study finds inconsistent correlations between the leaf and root traits of plants in temperate grassland communities due to different selective pressures on the aboveground and belowground components (Kembel and Cahill 2011). It is still unknown whether there is a correlation between the root and leaf traits of epiphytic plants whose roots and leaves are exposed to the atmosphere.

There is an increasing number of literatures on epiphytes, covering various aspects of their ecology, physiology, and anatomy (Zhang et al. 2012a, 2012b, Zotz and Winkler 2013, Zotz et al. 2014, Williams et al. 2020, De and Biswas 2022, Leão et al. 2023). Many studies suggested that there may be significant differences in anatomy between epiphytic and terrestrial life forms. For example, Schellenberger Costa et al. (2018) found that differences in functional traits across life forms are larger than differences within life forms along a large elevation gradient from savanna to alpine habitats. Epiphytes exhibited lower specific leaf area and leaf nitrogen content and greater trait variance than terrestrial species when experiencing stronger water and nutrient stresses along elevational gradients (Helsen et al. 2023). Li et al. (2017) observed that epiphytic orchids with a higher proportion of velamen radicum thickness show stronger drought resistance traits than terrestrial orchids. However, a comprehensive understanding of the differences in water-related characteristics between epiphytic and terrestrial species remains lacking.

The genus *Cymbidium*, belonging to the Orchidaceae family, has a distinctive ecological diversification and contains epiphytic, terrestrial, lithophytic, and saprophytic life forms (Yukawa and Stern 2002, Liu et al. 2006, Du Puy and Cribb 2007). This provides an ideal research system for understanding the water adaptation strategies of epiphytic plants and the correlations between root and leaf traits. In this study, we conducted a common garden experiment to evaluate leaf and root traits associated with water adaptation in 9 terrestrial and 15 epiphytic species in *Cymbidium*. Our key objectives were to examine the differences in root and leaf traits between closely related epiphytic and terrestrial species and to investigate the relationship between root and leaf traits related to water adaptation. We hypothesized that epiphytic and terrestrial *Cymbidium* species may exhibit distinct trait syndromes reflecting coordinated anatomical and physiological adaptations. Especially, epiphytes may have a greater capacity to adapt to low-moisture canopy environments, while terrestrial species may have a greater tolerance to waterlogging. In addition, the coordination between leaf and root traits in epiphytic species may be stronger than in terrestrial species, because the environments in which the roots and leaves of epiphytes are exposed are more similar.

## Materials and methods

### Study site and plant materials

We collected the samples of 24 *Cymbidium* species, including 9 terrestrial and 15 epiphytic species. The life forms of the tested species were defined based on the Flora of China (http://www.efloras.org), literature (Liu et al. 2006, Du Puy and Cribb 2007), and our field observations. In addition, our research aims to compare the water-related traits of species with significant moisture differences in habitats; thus, the facultative epiphytes and lithophytes with ambiguous water availability were excluded from our analysis. This collection was made in an orchid garden at Kunming Botanical Garden, Yunnan, China (elevation 1990 m, 102° 41′ E, 25° 01′ N). To ensure that observed interspecific differences were not due to variable growth conditions, the plants were cultivated for >12 months in a mixed media comprising of 10% humus and 90% bark (1 cm × 1 cm) in a greenhouse with an air temperature of 12–27°C and 45%–75% relative air humidity. Fertilizer was applied once a month, while the plants were watered every 3 days in summer and once a week in winter. The samples of newly produced, mature roots and leaves were taken from at least three different individuals per species. The leaves were obtained from their central region, carefully excluding the primary vein, while the roots were collected ∼2 cm above the tips of newly formed viable roots. These sampling and measurements were conducted from July to September, 2022.

### Measurement of root and leaf anatomy and morphology

To characterize the anatomical features of leaves and roots, the sampled leaves and roots were preserved in FAA (a 10:7:2:1 volume ratio of 95% alcohol, distilled water, formalin, and acetic acid) for no <24 h. After fixation, dehydration was carried out using an ethanol gradient, and the samples were embedded in paraffin for further sectioning. A rotary microtome (RM2126RT, Leica, Germany) was employed to obtain transverse sections. These tissues were analysed and captured through an optical microscope (DM2500, Leica). The parameters, including root velamen thickness (VT), root radius (R), root cortical cell length (CCL), root area (RA), root stele area (SA), root cortex area (CA), and leaf thickness (LT), were measured via ImageJ v.1.43u software (National Institutes of Health, Bethesda, MD, USA). The number of vessel (*N*_ves_) represents the count of primary xylem vessels. Both diameter of vessel (*D*_ves_) and area of vessel (*A*_ves_) were calculated by measuring all primary xylem vessels. The units and the functions of the tested leaf and root traits are given in Table 1.

**Table 1.** Leaf and root traits of the tested *Cymbidium* species.

Trait	Abbreviation	Unit	Function
Stomatal density	SD	mm^−2^	Gas exchange
Stomatal length	SL	μm	Gas exchange
Leaf thickness	LT	μm	Water conservation
Root velamen thickness	VT	μm	Water conservation
Root radius	R	mm	Water availability
Velamen thickness/radius	VT/R		Water conservation
Root cortical cell length	CCL	μm	Water storage
Number of vessel	*N*_ves_	No.	Water transport
Diameter of vessel	*D*_ves_	μm	Water transport
Area of vessel	*A*_ves_	μm^2^	Water transport
Cortex-to-stele ratio	CSR		Water storage and transport
Root cortex area	CA	mm^2^	Water storage
Root stele area	SA	mm^2^	Water transport
Root area	RA	mm^2^	Water availability
Stele-to-root ratio	SRR		Water transport
Cortex-to-root ratio	CRR		Water storage

For the evaluation of stomatal characteristics, the abaxial surfaces of the collected leaves were carefully peeled off. After drying, they were transferred to slides and then photographed using an optical microscope. The acquired images were analysed using the software ImageJ 1.43u (National Institutes of Health). Stomatal length (SL) was defined by the length of the guard cells, reflecting the maximum opening capacity of the stomata. Stomatal density (SD) was characterized as the number of stomata in each unit area of the leaf.

### Construction of phylogenetic tree

A phylogenetic tree depicting the relationships among these 24 *Cymbidium* species was developed using the plastome sequences shown in Table S1. *Acriopsis javanica* was selected as the outgroup due to its close relationship with *Cymbidium* (Zhang et al. 2022). The process of multiple alignments and trimming was conducted automatically by Phylosuite v.1.2.3 (Zhang et al. 2020). Phylogenetic analyses were carried out for each matrix employing the maximum likelihood approach on the CIPRES Science Gateway (http://www.phylo.org/) online tool RAxML v.8.2.12, illustrating the phylogenetic relationships and ecological information for the examined *Cymbidium* species (Fig. S1).

### Data analysis

To analyse the evolutionary patterns of leaf and root traits, we used the *K*-statistic to assess the phylogenetic signal of each trait, which relies on a ‘Brownian motion’ model (Blomberg et al. 2003). The significance is tested by comparing the variance of standardized contrasts to random values obtained by mixing trait data across the tips of the tree and indicates the difference between the sum of variation in a quantitative trait and its expected value. The *K*-metric measures phylogenetic conservatism, with values >1 indicating traits that are more conserved than expected under Brownian motion, while values <1 suggest that traits exhibit greater variability than anticipated from Brownian motion, reflecting significant lability (Blomberg et al. 2003). The *K*-statistic was performed using the ‘picante’ package v.1.8.2 in R v.4.2.3 (Kembel et al. 2010), revealing that all traits exhibited a weak signal of phylogeny (Table 3). Consequently, we ignored phylogenetic effect in the subsequent analyses.

The plasticity of the trait was evaluated through the coefficient of variation (CV), which is the ratio of the standard deviation to the mean multiplied by 100. In addition, all data were log_10_ transformed before analysis to improve normality and homoscedasticity. Student’s *t*-test was employed to compare traits between distinct groups. For each correlation coefficient (Table S2), *P*-values testing against the null hypothesis of no correlation were obtained using the corr.test function from the ‘psych’ package v2.5.3 in R (Revelle 2025), and linear fitting was performed using Origin 2018 SR1 (Origin Lab, Northampton, MA, USA). Principal component analysis (PCA) was conducted using Origin 2018 SR1.

### Compliance with ethical standards

This article does not contain any studies with human participants or animals performed by any of the authors.

## Results

Thirteen root traits and 3 leaf traits of 24 *Cymbidium* species were investigated in the present study. All traits were highly variable among *Cymbidium* species (Table 2). LT showed the greatest variation (CV = 74.54%), while the SD was also highly variable (30.80%). The SL showed the smallest variation (13.13%). Among the root traits, the root CA (56.43%) and the root cortex-to-stele ratio (CSR, 52.88%) varied greatly, while root CCL showed the smallest variation (15.69%).

**Table 2.** Variations and differences in leaf and root traits (mean ± SE) of 15 epiphytic and 9 terrestrial *Cymbidium* species.

Trait	Range	CV (%)	Epiphytic	Terrestrial	*P*-value
SD	49.97–185.06	30.80	125.86 ± 9.17	114.36 ± 13.87	.479^ns^
SL	18.37–36.45	13.13	26.18 ± 0.73	29.83 ± 1.25	.013*
LT	202.26–1441.04	74.54	420.60 ± 86.89	276.82 ± 15.27	.220^ns^
VT	147.67–930.47	31.12	616.48 ± 40.58	466.44 ± 56.10	.038*
R	1.52–2.76	17.34	2.04 ± 0.10	2.18 ± 0.12	.366^ns^
VT/R	0.05–0.42	32.19	0.31 ± 0.02	0.22 ± 0.03	.016*
CCL	83.55–147.81	15.69	100.16 ± 3.61	118.60 ± 5.05	.006**
*N*_ves_	9.33–17.67	15.88	13.35 ± 0.58	13.39 ± 0.68	.971^ns^
*D*_ves_	28.53–51.85	18.70	42.88 ± 1.46	32.10 ± 1.26	.000***
*A*_ves_	470.52–1660.86	36.59	1162.49 ± 80.17	675.15 ± 61.25	.000***
CSR	5.98–39.77	52.88	15.46 ± 2.27	26.75 ± 3.11	.007**
CA	2.78–19.96	56.43	5.71 ± 0.74	8.98 ± 1.57	.045*
SA	0.17–0.78	42.54	0.42 ± 0.04	0.38 ± 0.07	.586^ns^
RA	7.25–23.92	34.48	13.47 ± 1.29	15.28 ± 1.58	.390^ns^
SRR	0.01–0.06	44.72	0.030 ± 0.0035	0.025 ± 0.0040	.102^ns^
CRR	0.26–0.86	27.66	0.41 ± 0.03	0.57 ± 0.04	.004**

Abbreviations for leaf and root traits are given in Table 1. Statistical differences are assessed by Student’s *t*-test, and significant levels are expressed as follows: ***P ≤ 0.001, **P ≤ 0.01, *P ≤ 0.05, ^ns^*P* ≥ 0.05 respectively.

Habitat had an important effect on the leaf and root traits of *Cymbidium* species. Nine out of the 16 root and leaf traits were significantly different between terrestrial and epiphytic species (Table 2). Terrestrial species had a significantly larger SL than epiphytic species. SD and LT were smaller in terrestrial species than in epiphytic species, although the differences were not statistically significant. In terms of root traits, the values for CCL, CSR, CA, and root cortex-to-root ratio (CRR) of terrestrial species were significantly greater than those of epiphytic species, while the root VT, VT/R, diameter of vessel (*D*_ves_), and area of vessel (*A*_ves_) were significantly smaller in terrestrial species.

**Figure 1.**
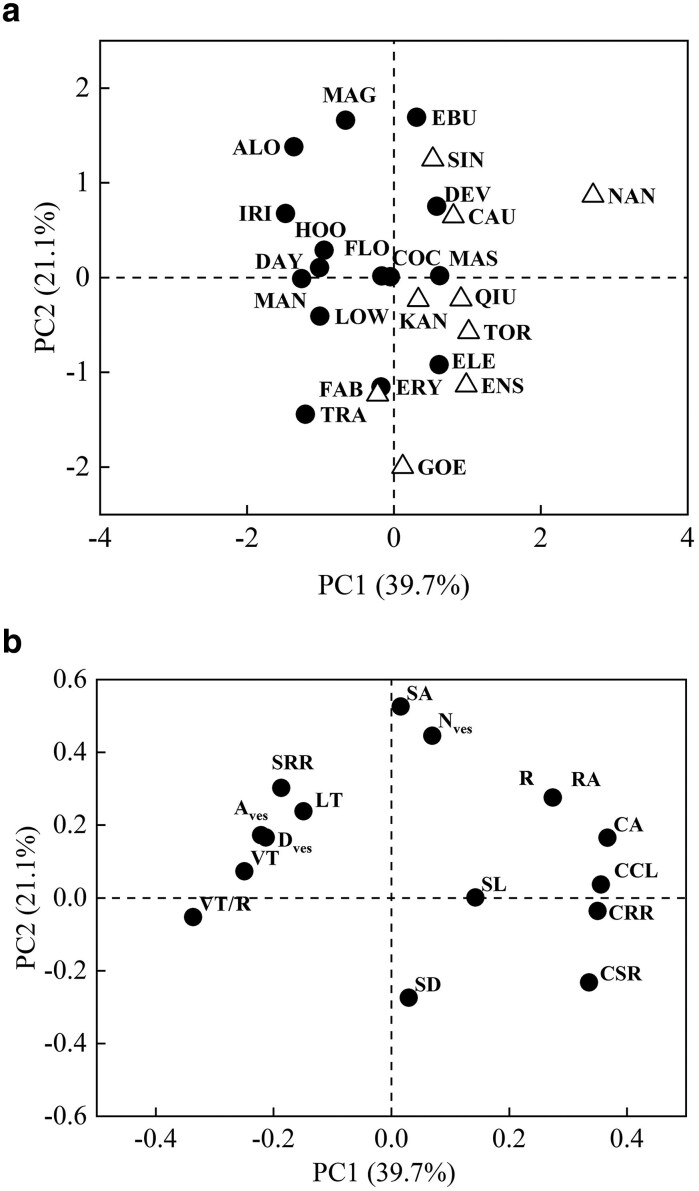
PCA based on species trait values (a) for 13 root traits and 3 leaf traits in 24 *Cymbidium* species. Loadings along PCA axes are presented in (b). Circles and triangles represent epiphytic and terrestrial *Cymbidium* species, respectively. FAB, *Cymbidium faberi*; GOE, *Cymbidium goeringii*; QIU, *Cymbidium qiubeiense*; CAU, *Cymbidium caulescens*; ENS, *Cymbidium ensifolium*; TOR, *Cymbidium tortisepalum*; SIN, *Cymbidium sinense*; KAN, *Cymbidium kanran*; NAN, *Cymbidium nanulum*; FLO, *Cymbidium floribundum*; HOO, *Cymbidium hookerianum*; LOW, *Cymbidium lowianum*; ERY, *Cymbidium erythraeum*; COC, *Cymbidium cochleare*; ELE, *Cymbidium elegans*; DEV, *Cymbidium devonianum*; DAY, *Cymbidium dayanum*; MAG, *Cymbidium maguanense*; MAS, *Cymbidium mastersii*; EBU, *Cymbidium eburneum*; IRI, *Cymbidium iridioides*; TRA, *Cymbidium tracyanum*; ALO, *Cymbidium aloifolium*; MAN, *Cymbidium mannii*. Abbreviations for leaf and root traits are given in Table 1.

The PCA results for species trait values revealed that the total variation was accounted for by 39.7% from the first principal component and 21.1% from the second principal component (Fig. 1a). The first PC axis showed a positive correlation with root radius (R), CCL, CSR, CA, RA, and CRR, but it was negatively correlated with VT, VT/R, *D*_ves_, and *A*_ves_. The second PC axis exhibited a positive correlation with R, *N*_ves_, SA, RA, and stele-to-root ratio (SRR; Fig. 1b). Species loadings revealed clear segregation of the two life forms along PC axis 1, with terrestrial and epiphytic species forming distinct clusters.

Our analysis of phylogenetic signals using the *K*-statistic (Table 3) revealed that all tested traits exhibited a weak signal of phylogeny. This result indicated environmental selection predominates over evolutionary constraints in shaping these functional characteristics, particularly in R (*K* = 0.208, *P* = .01), CCL (*K* = 0.189, *P* = .013), *D*_ves_ (*K* = 0.150, *P* = .04), CA (*K* = 0.265, *P* = .001), RA (*K* = 0.208, *P* = .007), and CRR (*K* = 0.166, *P* = .033).

**Table 3.** Phylogenetic signals of leaf and root traits in 24 *Cymbidium* species using Blomberg’s *K* statistics.

Trait	Phylogenetic signal	
	*K*-statistic	*P*-value
SD	0.132	.111
SL	0.025	.953
LT	0.136	.271
VT	0.123	.185
R	0.208	.01**
VT/R	0.186	.086
CCL	0.189	.013*
*N*_ves_	0.061	.621
*D*_ves_	0.150	.04*
*A*_ves_	0.117	.123
CSR	0.112	.146
CA	0.265	.001***
SA	0.091	.293
RA	0.208	.007**
SRR	0.063	.528
CRR	0.166	.033*

Significant correlations are shown in boldface. Asterisks denote significant levels: ***P ≤ 0.001, **P ≤ 0.01, *P ≤ 0.05, respectively.

**Figure 2.**
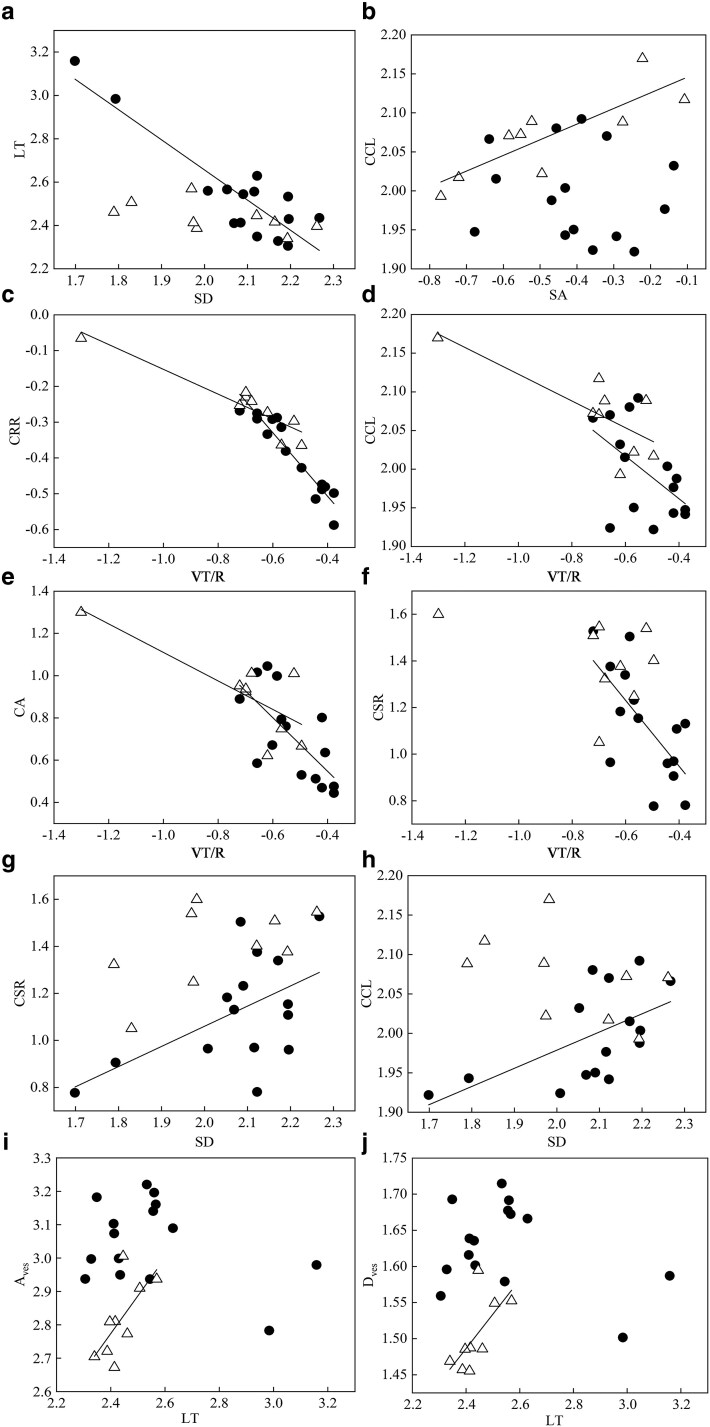
Correlations between leaf and root traits across 24 *Cymbidium* species. (a) SD in relation to LT for epiphytic (*r* = −0.893***) and terrestrial (*r* = −0.571) species. (b) Root SA to root CCL for epiphytic (*r* = −0.135) and terrestrial (*r* = 0.829**) species. (c–f) VT/R to CRR, CCL, root CA, and CSR for epiphytic (*r* = −0.947***, −0.527*, −0.688**, −0.674**, respectively) and terrestrial (*r* = −0.928***, −0.759*, −0.780*, −0.362, respectively) species. (g and h) SD in relation to CSR and root CCL for epiphytic (*r* = 0.546*, 0.585*, respectively) and terrestrial (*r* = 0.542, −0.476, respectively) species. (i and j) LT to area of vessel (*A*_ves_) and diameter of vessel (*D*_ves_) for epiphytic (*r* = −0.332, −0.324, respectively) and terrestrial (*r* = 0.682*, 0.664*, respectively) species. Circles and triangles represent epiphytic and terrestrial *Cymbidium* species, respectively. *P*-values testing against the null hypothesis of no correlation between traits are calculated using corr.test in R, and non-significant correlations are shown as data points without regression lines. *P ≤ .05; **P ≤ .01; ***P ≤ .001.

For leaf traits, a negative correlation was observed between SD and LT in epiphytic species (*r* = −0.893; Fig. 2a). For root traits, SA showed a positive correlation with CCL in terrestrial species (*r* = 0.829), but a negative correlation in epiphytic species (*r* = −0.135; Fig. 2b). On the other hand, both epiphytic and terrestrial species showed negative correlations between the traits related to root velamen and cortex (Fig. 2c–e). For example, VT/R showed negative correlations with water storage traits (CRR, CCL, and CA). In addition, a negative correlation was found between VT/R and CSR (*r* = −0.674) in epiphytic species only (Fig. 2f). Interestingly, in epiphytic species, SD related to leaf water loss was positively correlated with CSR (*r* = 0.546) and CCL (*r* = 0.585) related to water storage (Fig. 2g and h), while in terrestrial species, LT was positively correlated with *A*_ves_ and *D*_ves_ (*r* = 0.682 and 0.664, respectively) related to water transport (Fig. 2i and j).

## Discussion

This study revealed significant differences in root and leaf anatomical traits between closely related epiphytic and terrestrial *Cymbidium* species, with distinct trait correlations between life forms. These findings align with known physiological divergences, confirming epiphytes’ specialized adaptations to water-limited environments (Li et al. 2017, Qi et al. 2020, Ai et al. 2023).

While epiphytic habitats promote species diversity (Gravendeel et al. 2004), water scarcity remains their primary constraint (Zotz and Hietz 2001). Our analyses demonstrate that life form significantly influences velamen- and cortex-related traits. The velamen radicum structure, which consists of dead cells at maturity and features an internal exodermis, exhibits functional optimization through increased thickness, enhancing rapid water conservation during transient precipitation events (Dycus and Knudson 1957, Zotz and Winkler 2013), consistent with arid-adapted species patterns (Sanford and Adanlawo 1973). Regarding the cortex, a large cross-sectional area of porous cortex facilitates internal O_2_ diffusion and supports the extension of roots into anoxic soils (Sundgren et al. 2018). A previous study suggested that greater cortical thickness is associated with greater hydraulic resistance (Kong et al. 2017). Terrestrial *Cymbidium* species are found in the understorey, besides streams and valleys, and are frequently subjected to waterlogging (Liu et al. 2006). Thus, the observed larger cortex can enable it to adapt and survive in such an environment. In addition, variation in xylem vessel diameter is one of the most critical parameters for evaluating plant–water relations (Hacke et al. 2016). Epiphytic species exhibited larger xylem vessel diameters and areas, suggesting enhanced water transport efficiency compared with their terrestrial counterparts.

Regarding stomatal characteristics, reduced SD in epiphytes aligns with previous reports of lower values for SD, *D*_vein_, and *T_r_* compared with terrestrial species (Rada and Jaimez 1992). Stomata are essential channels for gas and water exchange within the plant body (Buckley 2019). Under water stress, plants can improve water use efficiency by adjusting stomatal size and density (Wang et al. 2007, Bertolino et al. 2019). Water shortage leads to small and dense stomata, which enables leaves to diffuse and transmit water under well-watered conditions quickly and to respond more rapidly to environmental stimuli under drought conditions, thereby enhancing water use efficiency (Zhang et al. 2012a, 2012b).

The observed synergies between water-related root and leaf traits support our hypothesis of trait interdependence. In epiphytes, positive correlations between SD and both CCL and CSR suggest enhanced root–leaf coordination, where water stored in cortical cells improves leaf water retention. For terrestrial species, the positive association between LT and vessel dimensions (*D*_ves_, *A*_ves_) indicates efficient root-to-shoot water transfer (von Arx et al. 2012), with increasing root water transport improving leaf water balance.

Notable intra-organ correlations emerged in epiphytes: the negative LT–SD relationship reflects hydraulic balance between CO_2_ assimilation need and diffusion resistance (Tanaka and Shiraiwa 2009, Zhang et al. 2012a, 2012b). Similarly, the negative VT/R–CSR relationship suggests coordinated root responses to the environment. These stronger relationships in epiphytic species, compared with terrestrial ones, result from their leaves and roots being in a similar environment. In contrast, the habitats of terrestrial species are less exposed to drought stress, so such trait interactions do not occur. This morphological construction illustrates the trade-off between the acquisition of resources and utilization (Baraloto et al. 2010).

Collectively, our study suggested that epiphytic and terrestrial species adopt different adaptive strategies according to their structural characteristics. The anatomical features of the epiphytic *Cymbidium* species adopt the strategy of ‘rapid water uptake—rapid water transport—effective water retention’, consisting of high VT, high *D*_ves_, high *A*_ves_, and low SL, whereas terrestrial species choose to build a higher CSR and larger cortical cells, which can facilitate the diffusion of O_2_ to the root tip and supply oxygen to the root system.

## Conclusion

The present study showed significant differences in leaf and root traits between epiphytic and terrestrial species of *Cymbidium*. In addition, species in different life forms employed different strategies and patterns in the interaction of their leaves and roots, with epiphytic species displaying greater potential for drought tolerance and terrestrial species showing stronger potential tolerance for waterlogging. Our study improves the understanding of how epiphytic and terrestrial species adapt through leaf and root traits to cope with their respective environments. The findings are important for understanding the ecological adaptation of orchids and promoting species conservation.

## Supplementary Material

plaf030_Supplementary_Data

## Data Availability

All data used in this study are available at https://osf.io/g7ez8/ and in the Supplementary data.
