# Supplementary material for: Differentiation in water adaptation strategy between epiphytic and terrestrial species of Cymbidium, Orchidaceae
Source: AoB Plants. 2025 Jun 2;17(4):plaf030. doi: 10.1093/aobpla/plaf030 (PMC12233011; doi:10.1093/aobpla/plaf030)
Supplement: plaf030_Supplementary_Data [file plaf030_supplementary_data.pdf]

Table S1 Plastome sequences used in phylogenetic analysis across 24 *Cymbidium* species.

Species	Voucher	Genbank accessions in NCBI
C. aloifolium	ZL324	OP080359
C. mannii	19HT2769	OP080412
C. dayanum	ZL23	ON983904
C. floribundum	18HT1234	OP080397
C. devonianum	ZL17	ON983906
C. tracyanum	ZL2	OP080407
C. iridioides	010-WBY	ON969305
C. erythraeum	19236	OP080504
C. hookerianum	O-1466	OP080388
C. lowianum	19HT2767-2	OP080410
C. eburneum	ZL27	OP080471
C. maguanense	ZL10	ON983928
C. mastersii	E00788025	ON983930
C. elegans	19HT2768	OP080514
C. cochleare	ZL25	OP080510

C. ensifolium	ZL442	OP080455
C. sinense	ZL4	OP080423
C. kanran	H3602	OP080479
C. qiubeiense	19HT2776	OP080355
C. goeringii	16281	OP080513
C. tortisepalum	ZL449	OP080430
C. faberi	18HT1855	OP080416
C. nanulum	2019/11/1②	OP080498
C. caulescens	H4603	OP080375
Acriopsis javanica	CYMW5	OP142286

Table S2 Coefficients of Pearson's correlations among leaf and root traits.

	SL	SD	LT	VT	R	VT/R	CCL	Nves	Dves	Aves	CSR	CA	SA	RA	SRR	CRR
SL		0.235	0.374	0.078	0.118	0.019	0.153	-0.097	0.395	0.423	0.208	0.075	-0.104	0.118	-0.237	-0.014
SD	-0.3		-0.571	-0.042	-0.47	0.11	-0.476	-0.614	-0.103	-0.043	0.542	-0.371	**-0.801****	-0.47	-0.665	-0.107
LT	0.284	**-0.893*****		0.568	0.459	0.346	0.311	0.006	**0.664***	**0.682***	-0.209	0.219	0.359	0.459	0.089	-0.228
VT	-0.128	0.039	-0.028		-0.266	**0.951*****	-0.56	-0.456	0.541	0.494	-0.274	-0.559	-0.383	-0.266	-0.283	**-0.869****
R	0.112	0.355	-0.2	0.393		-0.549	**0.869****	0.482	-0.091	0.042	0.369	**0.935*****	**0.656***	**1.000*****	0.027	0.565
VT/R	-0.204	-0.213	0.116	**0.773*****	-0.278		**-0.759***	-0.546	0.489	0.408	-0.362	**-0.780***	-0.535	-0.549	-0.246	**-0.928*****
CCL	-0.3	**0.585***	-0.47	-0.063	**0.669****	**-0.527***		0.518	-0.082	0.037	0.184	**0.953*****	**0.829****	**0.869****	0.366	**0.817****
Nves	0.093	-0.065	0.198	0.409	0.540*	0.032	0.024		-0.482	-0.51	-0.281	0.488	**0.722***	0.482	0.55	0.36
Dves	0.408	0.376	-0.324	0.241	0.308	0.028	-0.019	0.4		**0.976*****	-0.192	-0.244	-0.099	-0.091	-0.055	-0.419
Aves	0.404	0.292	-0.332	0.249	0.231	0.09	-0.09	0.343	**0.961*****		-0.054	-0.102	-0.067	0.042	-0.124	-0.304
CSR	0.113	**0.546***	-0.449	-0.337	0.481	**-0.674****	**0.765*****	-0.305	-0.049	-0.087		0.409	-0.364	0.369	**-0.793***	0.358
CA	0.234	0.343	-0.202	-0.084	**0.871*****	**-0.688****	**0.742****	0.347	0.191	0.123	**0.734****		**0.699***	**0.935*****	0.139	**0.822****
SA	0.182	-0.34	0.369	0.358	0.432	0.063	-0.135	**0.876*****	0.328	0.293	-0.47	0.251		0.656	**0.772***	0.57
RA	0.112	0.355	-0.199	0.393	**1.000*****	-0.278	**0.669****	**0.540***	0.308	0.231	0.481	**0.871*****	0.432		0.027	0.565
SRR	0.071	**-0.651****	**0.536***	-0.021	-0.51	0.317	**-0.745*****	0.338	0.029	0.066	**-0.892*****	**-0.563***	**0.556***	-0.51		0.279
CRR	0.297	0.157	-0.106	**-0.739****	0.254	**-0.947*****	0.484	-0.106	-0.074	-0.095	**0.742****	**0.697****	-0.137	0.254	-0.364	

Lower diagonal, epiphytic species; Upper diagonal, terrestrial species. Significant correlations are shown in boldface. Asterisks denote significant levels: ***, $P \leq 0.001$; **, $P \leq 0.01$; *, $P \leq 0.05$, respectively. See **Table 1** for definitions of abbreviations.

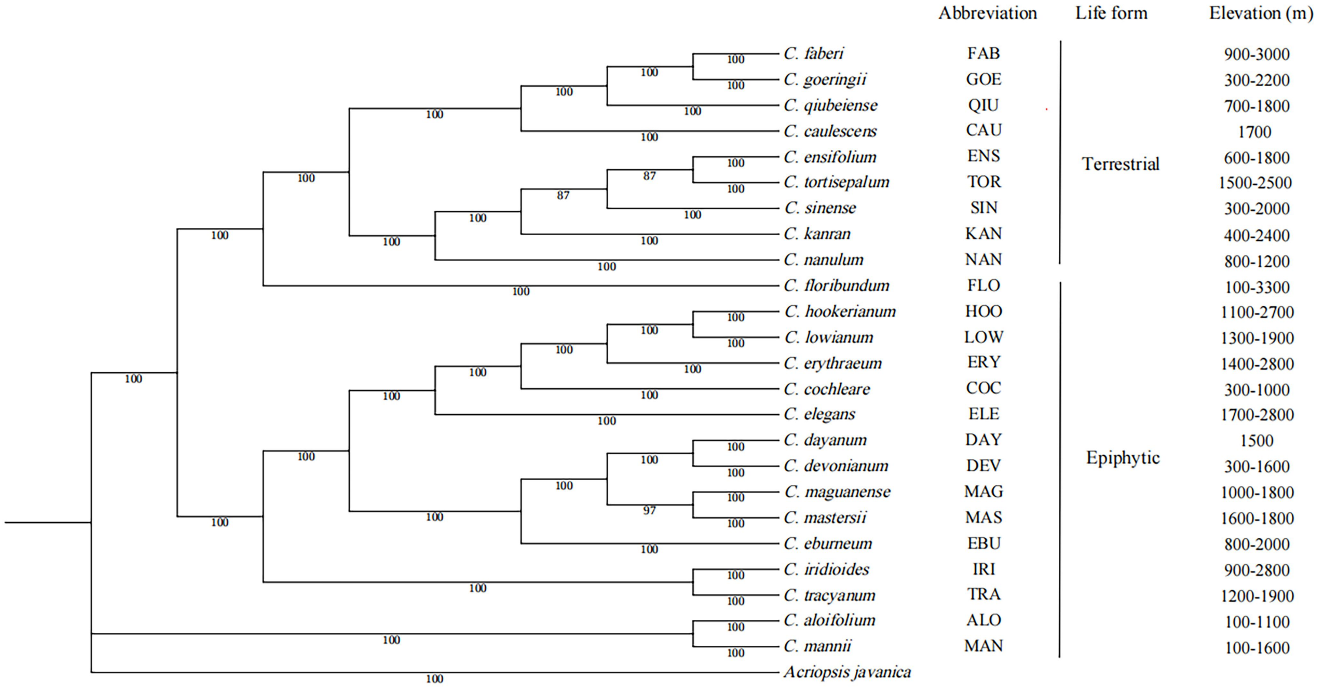

Fig. S1 Phylogenetic relationships and ecological information across 24 *Cymbidium* species. Numbers associated with nodes are the maximum-likelihood bootstrap value.
